# Changes of migraine aura with advancing age of patients

**DOI:** 10.1186/s10194-023-01642-w

**Published:** 2023-08-01

**Authors:** Adrian Scutelnic, Hristina Drangova, Antonia Klein, Nedelina Slavova, Morin Beyeler, Julian Lippert, Norbert Silimon, Thomas R. Meinel, Marcel Arnold, Urs Fischer, Franz Riederer, Heinrich P. Mattle, Simon Jung, Christoph J. Schankin

**Affiliations:** 1 Department of Neurology, Inselspital, Bern University Hospital, University of Bern, Freiburgstrasse, CH-3010 Bern, Switzerland; 2 Department of Neurology and Stroke Centre, University Hospital Basel and University of Basel, Basel, Switzerland

**Keywords:** Cortical spreading depression, Migraine with aura, Differential diagnosis of migraine with aura and ischemic stroke in elderly

## Abstract

**Aim:**

Given the similar presentation of migraine aura and acute ischemic stroke, advancing patient age might change the characteristics of migraine with aura (MA) and be clinically important. Clinical data, however, are limited. Experimental studies indicate a decrease in the magnitude of cortical spreading depression (CSD), the pathophysiological correlate of migraine aura, with advancing age. Our study aimed to assess the influence of age on the clinical features of MA.

**Methods:**

Three hundred and forty-three patients were interviewed using a structured questionnaire. The questions covered the headache characteristics and symptom types including the characteristics of the C-criterion, as defined by the International Classification of Headache Disorders 3^rd^ Edition. The association of age with MA characteristics was assessed.

**Results:**

The median age was 29 (IQR 28–52) and 235 of the 343 patients were women (69%). Individual symptoms of the C-criterion such as gradual aura spreading over longer than 5 min (*P* < 0.001), two or more aura symptoms occurring in succession (*P* = 0.005), duration of at least one MA symptom for longer than 60 min (*P* = 0.004), and associated headache (*P* = 0.01) were more frequent in younger patients. The number of symptoms (*P* = 0.003) including the C-characteristics decreased with increasing age (*P* < 0.027). Patients with sensory (*P* < 0.001), motor (*P* = 0.04) and speech disturbance (*P* = 0.02) were younger, and older patients with headache had less photophobia (*P* = 0.04) and phonophobia (*P* = 0.03). Sensitivity analyses yielded similar results.

**Conclusion:**

The frequency of typical characteristics of migraine aura and migraine headache including photophobia and phonophobia decreases with advancing patient age. This might have potentially difficult implications for the diagnosis of MA in the elderly.

**Supplementary Information:**

The online version contains supplementary material available at 10.1186/s10194-023-01642-w.

## Introduction

Migraine with aura (MA) symptoms are defined by the current diagnostic criteria [1] and include, for the aura, slow development, duration of 5–60 min, complete reversibility, positive irritative focal neurological symptoms, and accompaniment by headache within one hour of symptom onset. However, clinical observations showed that some patients with migraine aura do not have headache [2–4] and that an individual aura symptom might last longer than the 60 min allowed by the current criteria [5, 6]. The canonical cortical spreading depression (CSD) represents a slowly occurring and spreading depolarization across the cortex that is assumed to be the pathophysiological explanation for migraine aura [7]. Although CSD fits many of the clinical features of MA, it does not explain the full picture, especially not the clinical variability.

Experimental studies in rats showed that older age correlated negatively with CSD propagation [8]. However, there are limited clinical data on the influence of age on MA symptoms in humans. Aiba et al. [2] report a biphasic age-distribution of MA without headache. Donnet et al. [9] found that new onset MA in patients over 50 was not associated with headache or was accompanied more often by headache with non-migrainous features, and Hansen et al. describe less nausea with advancing age [10]. By contrast, Viana et al. did not find age to be a significant factor associated with prolonged aura in univariate analysis [11]. There is limited information on the influence of age on the individual symptoms of migraine aura.

The aim of this study was to assess the impact of age on MA symptoms. We hypothesized that the number of typical migraine features in MA decreases with advancing age.

## Methods

This monocentric cross-sectional study was approved by the local ethics committee (2018–02258). The methods were previously reported [12]. In short, patients with MA diagnosed according to the International Classification of Headache Disorders 3^rd^ edition (ICHD-3) [1] were interviewed using a structured questionnaire. The questions covered the type of symptoms, temporal aspects such as symptom onset and duration, the succession of symptoms, headache characteristics of a usual MA attack, as well as co-morbidities. Inclusion criteria for the study were verified diagnosis of MA, age more than 18 years and last migraine attack less than 12 months prior to the interview.

### Statistical analyses

Frequencies are presented as counts, crude odds ratios and confidence intervals, calculated using Wilson’s method. Continuous variables are presented as medians with interquartile range (IQR). For univariate comparisons of categorical variables ${x}^{2}$ or Fisher’s exact were used, as appropriate. For comparisons of continuous variables, the Mann–Whitney or Kruskal–Wallis test was used, as appropriate. For multiple comparisons, Bonferroni correction has been performed. A *P* value of < 0.05 was considered significant. Further, the association between the number of symptoms and age was calculated. Only visual, sensory, motor and speech symptoms were considered, given their well-defined cortical distribution. Given that the influence of acute or preventative therapy on headache was not assessed, headache frequency, intensity and duration were not included in the analysis. Specifically, we looked at the temporal succession of headache and aura, headache location, headache character, aggravation of headache by routine physical activity, nausea, photo- and phonophobia.

#### Sensitivity analyses

As sensitivity analyses univariate comparisons between patients younger and older than 60 years were performed. Furthermore, the interaction between C-characteristics, age per 10 years and sex was assessed using multivariable logistic regression. To assess the goodness of fit of the prediction model, the Pearson ${x}^{2}$ test was performed. Additionally, we performed post-hoc power analyses for all outcomes with a significance level of alpha of 0.05. For outcomes consisting of comparisons between two groups, we used the mean differences, for three or more groups the one-way ANOVA test. A power of at least 0.8 was considered sufficient. The statistical analyses were performed in STATA/MP 16.0, StataCorp LLC.

## Results

**Fig. 1:**
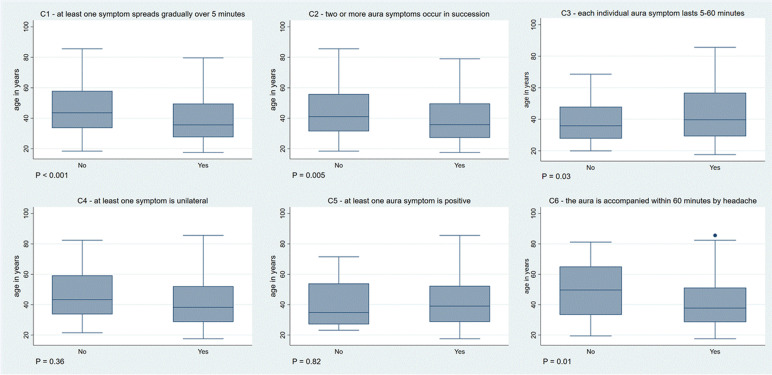
Association between each C-characteristic and age

Three hundred and forty-three patients were interviewed. The median age was 29 (IQR 28–52) and 235 of the 343 patients were women (69%). The interviews took place after a median of 28 days (IQR 7–118) after the last MA attack. For an overview of the association of each C-characteristic and age see Fig. 1.

### C-characteristics

#### C1-characteristic of the C-criterion – at least one symptom spreads gradually over 5 min

The C1 characteristic was fulfilled in 245/343 (71%). Those with fulfilled C1-characterstic were younger (median age 36 years [IQR 28–50] vs 44 [34–58], *P* < 0.001). In 205/343 (60%) patients at least one symptom spread either suddenly or over less than 5 min and in 15 (4%) over longer than 60 min. Although those with at least one slow-spreading symptom (i.e. > 60 min) were younger, the difference was not significant (34 years [IQR 26–51] vs 39 [29–53], *P* = 0.23). Patients with at least one symptom that spread < 5 min had a similar age compared with those with symptom onset of > 5 min (39 years [30–52] vs 38 [27–53], *P* = 0.73).

#### C2-characteristic of the C-criterion—two or more aura symptoms occur in succession

The C2-characteristic of the C-criterion was fulfilled in 201/343 (59%). Those with fulfilled C2-characteristic were younger (median age 36 years [IQR 27–59] vs 41 [IQR 31–56], *P* = 0.005). Looking only at patients with at least two symptoms (211/343, 61.5%), the difference in the median age of those with and without fulfilled C2-characteristic was not significant (36 years [27–50] vs 33 [28–45], *P* = 0.73).

#### C3-characteristic of the C-criterion – each individual aura symptom lasts 5–60 min

The C3-characteristic of the C-criterion was fulfilled in 211/343 (62%). Those with fulfilled C3-characteristic were older (median age 40 years [IQR 29–57] vs 36 [28–48], *P* = 0.03).

One hundred four of 343 (30%) had at least one symptom that lasted longer than 60 min and 17 (5%) less than 5 min. Patients with symptoms lasting > 60 min were younger compared to those with symptoms lasting < 60 min (35 years [27–47] vs 40 [30–56], *P* = 0.004).

#### C4-characteristic of the C-criterion – at least one symptom is unilateral

The C4-characteristic was fulfilled in 327/343 (95%). There was no difference in median age between those with and without fulfilled C4-characteristic (38 years [IQR 29–52] vs 43[34–59], *P* = 0.36).

#### C5-characteristic of the C-criterion – at least one aura symptom is positive

The C5-characteristic was fulfilled in 323/343 (94%). There was no difference in median age between those with and without fulfilled C4-characteristic (median age 39 years [IQR 29–52] vs 35 [27–54], *P* = 0.82).

#### C6-characteristic of the C-criterion – the aura is accompanied, or followed within 60 min by headache.

The C6-characteristic was fulfilled in 315/343 (92%). Patients with headache were significantly younger (median age 38 years [IQR 29–51] vs 50 [33–65], *P* = 0.01). Looking at the headache characteristics, patients with photophobia (38 years [IQR 28–51]) were younger than those without (40 years [31–61], *P* = 0.04), as were those with phonophobia (37 years [IQR 28–51] vs 40 [31–57], *P* = 0.03). Other headache characteristics did not have an association with age (Table 1).

**Table 1:** Association of headache characteristics and age

**Headache characteristic**	**Characteristic not fulfilled, age median (IQR)**	**Characteristic fulfilled, age median (IQR)**	***P* – value (Mann-Whitney)**
Occurrence of aura before the headache onset	40 (31-55)	38 (28-51)	0.46
Occurrence of aura at the same time as the headache	39 (29-53)	35 (25-48)	0.06
Occurrence of aura after the headache onset	39 (29-53)	39 (31-50)	0.8
Unilateral headache location (vs bilateral)	40 (29-55)	37 (28-50)	0.12
Pulsating headache	39 (29-56)	39 (29-50)	0.4
Stabbing headache	38 (29-53)	40 (29-50)	0.98
Dull headache	39 (30-55)	37 (28-51)	0.55
Aggravation by routine physical activity	36 (29-57)	39 (29-50)	0.69
Nausea	39 (31-56)	38 (28-51)	0.27
Photophobia	40 (31-61)	38 (28-51)	0.04
Phonophobia	40 (31-57)	37 (28-51)	0.03

#### Number of fulfilled C-characteristics

**Fig. 2:**
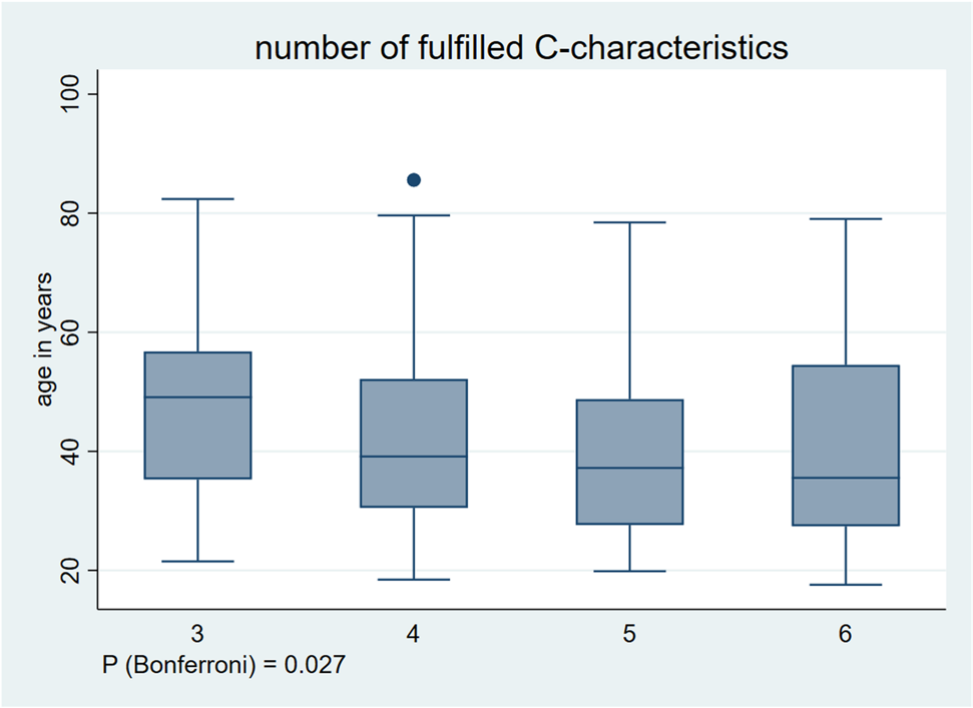
Association between number of fulfilled C-characteristics and age

There were 39 patients (11% of 343) with three, 94 (27%) with four, 131 (38%) with five and 79 (23%) with six fulfilled C-characteristics. The median age of patients who had fulfilled three C-characteristics was 49 [IQR 35–57], older than patients with four (39 [31–52]), five (37 [27–48]) and six fulfilled C-characteristics (36 [27–55]). The four groups differed significantly from each other (*P*_Bonferroni_ = 0.027, Fig. 2). In post-hoc analysis, there was a significant difference between the subgroups with three and four (*P* = 0.03), three and five (*P* = 0.001) and three and six characteristics (*P* = 0.009), but no difference between those with four and five (*P* = 0.13), four and six (*P* = 0.24) and five and six C-characteristics (*P* = 0.9).

### Number of symptoms

**Fig. 3:**
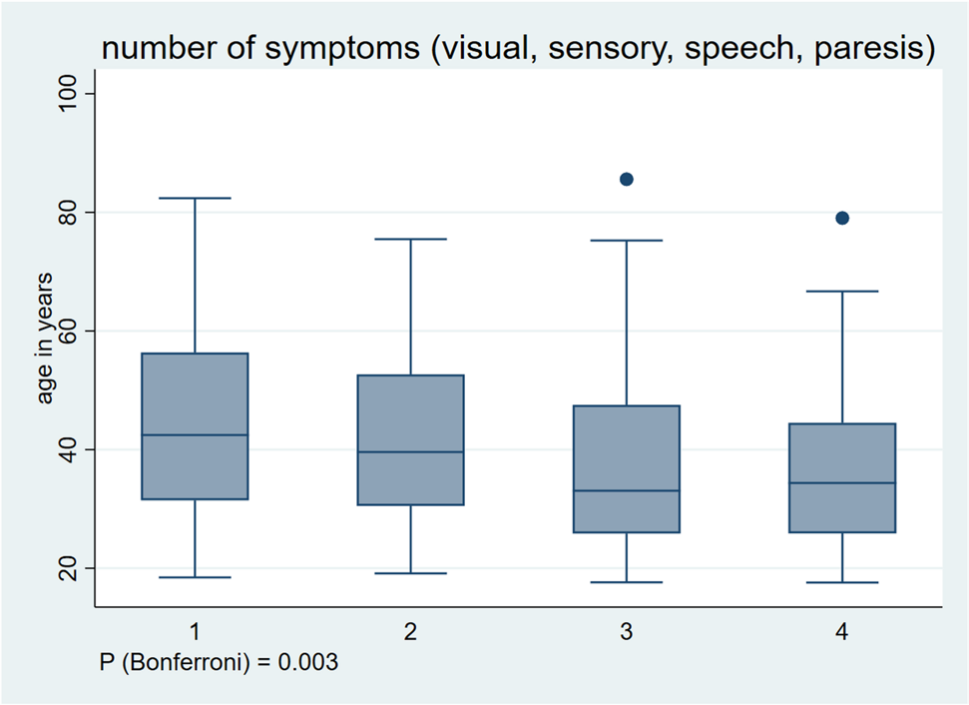
Association between number of symptoms and age

There were 134 patients (39%) with one, 87 (25%) with two, 84 (24%) with three and 38 (11%) with four symptoms. Patients with one (median age 42 years [IQR 31–56]) and two symptoms (median age 40 years [31–53]) were older than those with three (median age 33 years [26–48]) and four symptoms (median age 34 years [26–45]). The four groups differed significantly from each other (*P*_Bonferroni_ = 0.003, Fig. 3). When comparing the subgroups for post-hoc analysis, there was a significant difference in age between patients with one and three symptoms (*P* = 0.0007), one and four (*P* = 0.007) and two and three symptoms (*P* = 0.02) but no difference between those with two and four (*P* = 0.07) and three and four symptoms (*P* = 0.89).

### Type of symptoms

Patients with visual symptoms (*n* = 326, 95%) were older (median age 39 years [IQR 29–52]) than those without (*n* = 17, 5%) who were 35 years old in median [IQR 31–52], the difference not reaching significance (*P* = 0.73). Patients with sensory disturbance (*n* = 175, 51%) were significantly younger compared to those without (*n* = 166, 49%), i.e. median age of 34 years [IQR 27–48] vs 42 [32–57] (*P* < 0.001). Patients with paresis (*n* = 50, 15%) were also significantly younger (median age 34 years, [IQR 26–47]) compared to those without paresis (39 years [IQR 29–54]; *P* = 0.04) as were patients with speech disturbance (*n* = 160, 47%, median age 35 years [IQR 27–51]) compared to patients without (*n* = 183, 53%, median age 41 years [IQR 31–53], *P* = 0.02).

Of the patients without visual symptoms (*n* = 17), all (100%) had sensory, three (18%) had motor, and 7 (41%) had speech disturbance.

Of patients with paresis, 6/50 [12%] had bilateral paresis and there was no difference in age compared to those with unilateral paresis (median age 38 years [IQR 31–47] vs 34 [26–46], *P* = 0.42). Similarly, the age of the 22/50 (44%) patients with wandering motor symptoms was similar to those without (median 32 years [IQR 24–42] vs 36 [28–50], *P* = 0.17). The median age of patients with paresis lasting less than five minutes (*n* = 3/50 [6%], median age 33 years [IQR 23–50]) was similar to those with paresis lasting 5–60 min (*n* = 22/50 [44%], median age 33 years [IQR 25–47]) and 60 min to 24 h (17/50 [34%], median age 36 years [IQR 29–45]). However, patients with paresis lasting longer than 24 h were non-significantly younger than those without (*n* = 5/50 [10%], median age 26 years [IQR 26–31] vs 35 [30–48], *P* = 0.14). Only four patients (8% of 50) with paresis were older than 60 years.

### Sensitivity analyses

The differences in C-characteristics were confirmed in our sensitivity analysis comparing people younger to older than 60 years old (Table 2), even after adjustment for sex (Tables 2 and 3).

**Table 2:** C-characteristics stratified by age

**C-characteristic**	**Age < 60 years (*N* = 295)**	**Age > 60 years (*N* = 48)**	**Unadjusted OR (95%CI)**	***P* value for uOR**	Adjusted^*^ OR (95%CI)	*P* value for adjusted^*^ OR
C1**-**at least one symptom spreads gradually over 5 min, n/N (%)	219 (74)	26 (54)	2.43 (1.23–5)	0.004	2.49 (1.33–5)	0.004
C2**-**two or more aura symptoms occur in succession, n/N (%)	182 (62)	19 (40)	2.45 (1.26–5)	0.004	2.43 (1.30–5)	0.005
C3**-**each individual aura symptom lasts 5–60 min, n/N (%)	171 (58)	40 (83)	0.27 (0.1–0.62)	< 0.001	0.27 (0.12–0.61)	0.001
C4**-**at least one symptom is unilateral, n/N (%)	283 (96)	44 (92)	2.14 (0.48–7)	0.19	2.14 (0.66–7)	0.20
C5**-**at least one aura symptom is positive, n/N (%)	277 (94)	46 (96)	0.67 (0.72–2.95)	0.60	0.65 (0.14–2.92)	0.58
C6**-**the aura is accompanied, or followed within 60 min by headache, n/N (%)	278 (94)	37 (77)	4.86 (1.89–12)	< 0.001	4.79 (2.07–11)	< 0.001

^*^OR adjusted for sex

**Table 3:** Multivariable logistic regression for the C-characteristics and age (per 10 years) adjusted for sex

**C-characteristic**	**aOR (95%CI)**	***P* value for aOR**
C1**-**at least one symptom spreads gradually over 5 min	0.76 (0.65-0.88)	0.0005
C2**-**two or more aura symptoms occur in succession	0.80 (0.69-0.92)	0.003
C3**-**each individual aura symptom lasts 5–60 min	1.24 (1.06-1.44)	0.0047
C4**-**at least one symptom is unilateral	0.84 (0.62-1.15)	0.300
C5**-**at least one aura symptom is positive	1.03 (0.76-1.39)	0.814
C6**-**the aura is accompanied, or followed within 60 min by headache	0.68 (0.54-0.87)	0.002

Looking at the differences in headache characteristics between patients younger and older than 60 years (Table 4), the significances for photophobia and phonophobia remained. However, a higher proportion of younger patients reported pulsating headache (45% vs 25%, *P* = 0.01) and aggravation by routine physical activity (77% vs 56%, *P* = 0.002). There was no difference for nausea (66% vs 54%, *P* = 0.09).

**Table 4:** Characteristics of headache stratified by age

**Headache characteristic**	**Age < 60 years (*N* = 295)**	**Age > 60 years (*N* = 48)**	**Unadjusted OR (95%CI)**	***P* – value**
Occurrence of aura before the headache onset, n (%)	211 (72)	32 (67)	1.25 (0.6-2.5)	0.49
Occurrence of aura at the same time as the headache, n (%)	40 (14)	3 (6)	2.35 (0.69-12)	0.23
Occurrence of aura after the headache onset, n (%)	39 (13)	6 (13)	1.06 (0.41-3.27)	0.89
Unilateral headache location (vs bilateral), n (%)	172 (58)	34 (71)	0.57 (0.27-1.15)	0.10
Pulsating headache, n (%)	132 (45)	12 (25)	2.42 (1.17-5)	0.01
Stabbing headache, n (%)	65 (22)	9 (19)	1.22 (0.54-3.02)	0.61
Dull headache, n (%)	115 (39)	16 (33)	1.27 (0.64-2.60)	0.45
Aggravation by routine physical activity, n (%)	229 (77)	27 (56)	2.69 (1.35-5)	0.002
Nausea, n (%)	196 (66)	26 (54)	1.67 (0.85-3.2)	0.09
Photophobia, n (%)	249 (84)	32 (67)	2.70 (1.27-6)	0.003
Phonophobia, n (%)	222 (75)	29 (60)	1.99 (1.05-3.76)	0.03

With regard to the analyzed outcomes, the post-hoc analyses showed power levels of 1 for all C-characteristics, 0.95 for number of C-characteristics and 0.96 for number of symptoms (Supplemental Tables S1, S2 and S3).

The goodness of fit analyses with one degree of freedom showed *P* values higher than the predefined significance level of 0.05 and therefore the adjustment model for the logistic regression could not be rejected (Supplemental Table S4).

## Discussion

We analyzed the influence of age on the MA characteristics. The main findings of our study are: a) patients with gradually spreading symptoms over 5 min, symptoms occurring in succession, and symptoms lasting longer than 60 min were younger, b) patients with a higher number of aura symptoms were younger, c) patients with lack of headache were older and the headache, when present, was less often accompanied by photo- and phonophobia with advancing age, and d) patients with sensory symptoms, paresis or speech disturbance were younger.

The gradual onset of symptoms and symptoms occurring in succession are thought to be clinical expressions of the canonical CSD [1]. In this context, our results in a large group of patients fit the experimental findings in animals of a negative impact of older age on CSD propagation [8]. Similarly, the longer duration of aura symptoms in younger patients may correspond to a higher susceptibility to, or a longer duration of, CSD. It is plausible that the lower number of symptoms reported by older patients might reflect a smaller area of the cortex affected by CSD.

The association of older age with a lack of headache has been reported in previous studies [3, 4, 9, 13]. However, the precise reason for this association is not yet fully understood. The less intense CSD with advancing age might, at least in part, explain this finding. However, it is still debated which role CSD plays in the generation of migraine headache in patients with MA [7]. Curiously, with advancing age, less photo- and phonophobia were reported by those who did have headache with their aura, with patients younger than 60 years having more often pulsating headache and aggravation by routine physical activity. In our study, there was no significant difference in the prevalence of nausea based on age. However, it is worth noting that a non-significantly higher proportion of patients younger than 60 years experienced nausea with their episodes, in-line with previous reports [9, 10].

Overall, with advancing age, the MA tends to exhibit fewer typical features, and this has significant clinical implications. Considering that older patients are more prone to having vascular risk factors, there is a risk of misdiagnosing a migraine aura as an ischemic event, particularly if it occurs for the first time. The consequence may be unnecessary ancillary examinations and secondary prevention treatments. Conversely, an actual ischemic event might present with migraine-like symptoms, making it challenging or even impossible to differentiate between the two without further ancillary investigations [14–17].

In our study, we observed that patients with visual symptoms tended to be older. Therefore, even with advancing age, their presence should argue in favor of a migraine aura diagnosis. As previously discussed by Russel & Olesen, aura with sensory, motor and/or speech symptoms without visual symptoms should prompt suspicion of alternative etiologies [13]. Nevertheless, younger patients reported non-visual symptoms more frequently, which might make them less useful in the clinical differential diagnosis of MA in general. This is of particular importance given that younger patients are more likely to present with migraine-like symptoms during an ischemic stroke as well [18].

In our study, we included patients who met the ICHD-3 criteria for definite MA. Given the tendency of MA to become less typical for migraine aura with advancing age, it is possible that some cases of MA in older patients might have been overlooked.

Our study has limitations. First, we did not assess the intra-individual variability of the MA attacks. Second, the interview took place after a median of 28 days after the last attack with a large interquartile range (7–118), making our results susceptible to recall bias. However, patients with MA experience many attacks during their lifetime, which might reduce this problem. We did not assess whether the patients took treatment for their headache and therefore, we did not analyze the headache intensity and duration. Furthermore, we did not assess the use of migraine preventive medication, which could potentially influence the presentation and characteristics of MA. Also, we did not assess the socio-economic status, which may have an influence on the diagnosis and symptoms of MA [19, 20]. Lastly, the study design was cross-sectional, not allowing firm conclusions and further longitudinal studies are needed. The strengths are the large number of patients included and the assessment of symptoms using a personal interview and a structured questionnaire.

In conclusion, with advancing age, the characteristics of MA have fewer migraine-like features and the headache, when present, is accompanied by less migrainous features such as photo- and phonophobia. Our findings highlight the difficulties and challenges in the clinical diagnosis of MA with advancing age.

### Supplementary Information

**Additional file 1: Table S1.** Post-hoc power calculations for the comparisons of the C-characteristics. **Table S2.** Post-hoc power calculation for the number of fulfilled C-characteristics. **Table S3.** Post-hoc power calculation for the number of symptoms. **Table S4.** Goodness of fit calculations for adjustment model including sex and age for C-characteristics as outcomes.

## Data Availability

The datasets used and/or analyzed during the current study are available from the corresponding author on reasonable request.

## Abbreviations

MA: Migraine with aura
CSD: Cortical spreading depression
ICHD-3: International Classification of Headache Disorders 3^rd^ Edition
