# Correction: Changes of migraine aura with advancing age of patients

**DOI:** 10.1186/s10194-023-01647-5

**Published:** 2023-08-14

**Authors:** Adrian Scutelnic, Hristina Drangova, Antonia Klein, Nedelina Slavova, Morin Beyeler, Julian Lippert, Norbert Silimon, Thomas R. Meinel, Marcel Arnold, Urs Fischer, Franz Riederer, Heinrich P. Mattle, Simon Jung, Christoph J. Schankin

**Affiliations:** 1 Department of Neurology, Inselspital, Bern University Hospital, University of Bern, Freiburgstrasse, CH-3010 Bern, Switzerland; 2 Department of Neurology and Stroke Centre, University Hospital Basel and University of Basel, Basel, Switzerland

**Correction** **:** **J Headache Pain 24, 100 (2023)**

**https://doi.org/10.1186/s10194-023-01642-w**

Following publication of this article [1], the author group became aware of some errors in the Results section of the Abstract.

The correct values are given below in bold, and the original article has been corrected.

**Results**

The median age was 29 (IQR 28–52) and 235 of the 343 patients were women (69%). Individual symptoms of the C-criterion such as gradual aura spreading over longer than 5 min (P < 0.001), two or more aura symptoms occurring in succession (P = 0.005), duration of at least one MA symptom for longer than 60 min (P = 0.004), and associated headache (P = 0.01) were more frequent in younger patients. The number of symptoms **(P = 0.003)** including the C-characteristics decreased with increasing age **(P < 0.027)**. Patients with sensory (P < 0.001), motor **(P = 0.04)** and speech disturbance (P = 0.02) were younger, and older patients with headache had less photophobia (P = 0.04) and phonophobia (P = 0.03). Sensitivity analyses yielded similar results.
