# Supplementary material for: Changes of migraine aura with advancing age of patients
Source: J Headache Pain. 2023 Aug 1;24(1):100. doi: 10.1186/s10194-023-01642-w (PMC10394819; doi:10.1186/s10194-023-01642-w)
Supplement: Supplementary file 1 — Additional file 1: Table S1. Post-hoc power calculations for the comparisons of the C-characteristics. Table S2. Post-hoc power calculation for the number of fulfilled C-characteristics. Table S3. Post-hoc power calculation for the number of symptoms. Table S4. Goodness of fit calculations for adjustment model including sex and age for C-characteristics as outcomes. [file 10194_2023_1642_MOESM1_ESM.docx]

**Supplemental tables**

Table S1. Post-hoc power calculations for the comparisons of the C-characteristics

|  | Criterion fulfilled | Criterion not fulfilled | Power |
| --- | --- | --- | --- |
| C1, mean age (SD) | 40 (14) | 46 (17) | 1 |
| C2, mean age (SD) | 39 (14) | 44 (16) | 1 |
| C3, mean age (SD) | 43 (17) | 38 (12) | 1 |
| C4, mean age (SD) | 41 (15) | 45 (17) | 1 |
| C5, mean age (SD) | 41 (15) | 41 (15) | 1 |
| C6, mean age (SD) | 41 (15) | 50 (19) | 1 |

Table S2. Post-hoc power calculation for the number of fulfilled C-characteristics.

| Number of fulfilled C-characteristics | 3 | 4 | 5 | 6 | Power |
| --- | --- | --- | --- | --- | --- |
| Mean age (SD) | 48 (16) | 43 (16) | 39 (14) | 40 (16) | 0.96 |

Table S3. Post-hoc power calculation for the number of symptoms

| Number of symptoms | 1 | 2 | 3 | 4 | Power |
| --- | --- | --- | --- | --- | --- |
| Mean age (SD) | 45 (16) | 42 (14) | 37 (15) | 37 (14) | 0.95 |

Table S4. Goodness of fit calculations for adjustment model including sex and age for C-characteristics as outcomes

| **C - characteristic** | **Pearson ꭓ^2^ value for goodness of fit** | ***P* value for goodness of fit** |
| --- | --- | --- |
| C1 - at least one symptom spreads gradually over 5 minutes | 342.13 | 0.427 |
| C2 - two or more aura symptoms occur in succession | 338.31 | 0.485 |
| C3 - each individual aura symptom lasts 5-60 minutes | 337.68 | 0.494 |
| C4 - at least one symptom is unilateral | 343.69 | 0.404 |
| C5 - at least one aura symptom is positive | 343.03 | 0.413 |
| C6 - the aura is accompanied, or followed within 60 minutes by headache | 373.08 | 0.09 |
